# Blood glucose level affects prognosis of patients who received intravenous thrombolysis after acute ischemic stroke? A meta-analysis

**DOI:** 10.3389/fendo.2023.1120779

**Published:** 2023-04-12

**Authors:** Yue Wang, Guannan Jiang, Jie Zhang, Jingfeng Wang, Wanchun You, Juehua Zhu

**Affiliations:** ^1^ Department of Neurology, The First Affiliated Hospital of Soochow University, Suzhou, China; ^2^ Department of Neurosurgery and Brain and Nerve Research Laboratory, The First Affiliated Hospital of Soochow University, Suzhou, China; ^3^ Department of Neurology, The Second People’s Hospital of Kunshan, Suzhou, China

**Keywords:** blood glucose, acute ischemic stroke, intravenous thrombolytic therapy, functional outcome, symptomatic intracranial hemorrhage

## Abstract

**Background and objectives:**

Intravenous recombinant tissue plasminogen activator (rtPA) thrombolysis is an effective treatment for acute ischemic stroke. Hyperglycemia is a major risk factor for the occurrence, development, and prognosis of ischemic stroke. This meta-analysis purposefully estimates the association between hyperglycemia and poor prognosis in acute ischemic stroke patients receiving intravenous rtPA thrombolytic therapy.

**Materials and methods:**

According to the predefined inclusion criteria, we searched PubMed, Web of Science, and Cochrane Library databases. The association of high blood glucose(>140mg/dl) with symptomatic intracranial hemorrhage (sICH), poor clinical outcome and mortality at 90 days post-rtPA thrombolysis was studied using both a common effects model and a random effects model. Odds ratios (ORs) were plotted on forest plots.

**Results:**

Of a total cohort of 2565 patients who received intravenous thrombolytic therapy, 721 had higher blood glucose. High glucose level significantly increased the odds of sICH (OR 1.80; 95% confidence interval(95%CI): 1.30- 2.50) and poor clinical outcome at 90 days (OR 1.82; 95%CI: 1.52-2.19), and all-cause mortality at 90 days (OR 2.51; 95%CI:1.65-3.82).

**Conclusions:**

In our meta-analysis, high blood glucose was significantly associated with sICH, poor clinical outcome and higher mortality at 90 days.

## Introduction

Acute ischemic stroke (AIS) is a common cerebrovascular disease around the world, with the characteristics of high morbidity and mortality. Hence, prompt AIS treatment is extremely important. Commonly used treatments for AIS include intravenous thrombolysis treatment (IVT), endovascular interventions, as well as antiplatelet and fiber-lowering treatments. IVT, as a widely used treatment for AIS, is required to administer recombinant tissue plasminogen activator (alteplase) to patients within 4.5h or urokinase (UK) within 6h (1). Previous studies have found that IVT decreases morbidity and mortality of patients after AIS. IVT can dissolve the fibrin in the thrombus to achieve the purpose of unclogging blood vessels. However, IVT brings up risks of complications such as symptomatic intracranial hemorrhage (sICH), post-thrombolytic cerebral edema, and allergy (2). Among them, sICH is the most serious complication. According to the National Institute of Neurological Disorders and Stroke (NINDS) Stroke Intravenous Thrombolysis Study criteria, sICH was defined as any manifestation of clinical deterioration within 36 h after revascularization therapies like IVT, along with CT showing cerebral hemorrhage. Although sICH accounts for only 6% of post-thrombolytic complications, it consists of 50%~80% of mortality caused by post-thrombolytic complications (3, 4).

More than half of patients with ischemic stroke have hyperglycemia at the time of admission (5). Previous studies have found that clinical outcomes in patients with AIS undergoing IVT are associated with hyperglycemia on admission to hospital. It was found that hyperglycemia may partially offset the beneficial effects of early recovery blood flow by IVT (6). It has been convinced that patients receiving intravenous alteplase may suffer worse clinical outcomes if admission hyperglycemia occurs (7–10). Although IVT induced partial infarct recanalization, patients with higher blood glucose (blood glucose on admission > 140mg/dl) had worse outcomes and would be more likely to have worse functional outcomes, sICH and even death than those with non-high blood glucose (blood glucose on admission < 140mg/dl) (11–13). As a result, the effect of high blood glucose on prognosis of AIS patients after IVT has received considerable critical attention.

Therefore, we conducted this meta-analysis in order to clarify the relationship between blood glucose levels and the prognosis of patients with AIS after IVT. We analyzed the 3 main indicators after IVT including rate of sICH, modified Rankin Score(mRS) and mortality. With this study, we hope to provide new perspectives for future clinical research and treatment for AIS.

## Methods

The study was designed, conducted, and reported per the Preferred Reporting Items for Systematic Reviews and Meta-Analyses (PRISMA) statement (14, 15).

### Search strategies

We conducted article searches in PubMed, Web of Science, and Cochrane Library databases up to October 20, 2022. The search strategies were as follows: (“Blood Sugar” OR “Sugar, Blood” OR “Glucose, Blood”) AND (“Stroke” OR “Transient Ischemic Stroke” OR “TIA” OR “Cerebral Infarction” OR “Cerebrovascular Infarction”) AND (“Therapeutic Thrombolysis” OR “Therapeutic Thrombolyses” OR “Thrombolyses, Therapeutic” OR “Thrombolysis, Therapeutic” OR “Therapy, Fibrinolytic” OR “Fibrinolytic Therapies” OR “Therapies, Fibrinolytic” OR “Therapy, Thrombolytic” OR “Therapies, Thrombolytic” OR “Thrombolytic Therapies” OR “Fibrinolytic Therapy”). No language restriction was applied to the search of human studies. Additionally, we manually screened the references for possible related studies in the relevant original and review articles.

### Inclusion and exclusion criteria

Following the recommended PICOS criteria, we developed inclusion criteria according to the meta-analysis’ aim: (1) Patients had to be older than 18 years of age received IVT for AIS; (2) Patients were divided into high and non-high groups based on their admission blood glucose, and their cut-off value was set at 140 mg/dl. (3) The outcomes to be observed are the incidence of sICH, poor clinical outcome assessed by mRS at 90 days and mortality at 90 days in AIS patients treated with intravenous tissue plasminogen activator. A poor clinical outcome is defined as a mRS> 2 at 90 days (16). (4) The study design had to be a randomized controlled trial or a longitudinal observational study.

The exclusion criteria follow the following points: (1) A review, editorial, meta-analysis, studies enrolling patients with hemorrhagic stroke, and studies not analyzing blood glucose or reporting the outcomes of interest were excluded. (2) Grey literature, including conference abstracts and unpublished data, was excluded. Studies from these sources are not peer-reviewed, so including them in a meta-analysis might cause results to be inconsistent.

There were 2 authors who independently completed the database search and screening, data collection, and quality assessment of the study. If the 2 authors are in dispute, we will contact the corresponding author to discuss the results. We collected data on research information, diagnosis, definition of hyperglycemia, follow-up duration, outcomes when the associations between high blood glucose and outcomes of interest were presented. An assessment of study quality was conducted by using the Newcastle-Ottawa Scale (NOS), which included scoring relating to the selection criteria and comparability of the groups (17). The scale ranged from 1 to 9, with more stars indicating higher study quality. We considered studies that had long enough follow-up periods as those with a mean follow-up period of at least 3 months (90 days).

### Statistical analyses

We standardized risk factors across studies whenever possible in order to compare data from different studies. According to the original study, we accepted all criteria for risk factor categories. We extracted data on the rate of sICH incidence, occurrence rate of poor clinical outcomes (mRS > 2)and mortality in the high glucose and non-high glucose groups of patients with AIS receiving intravenous tissue plasminogen activator, and expressed the relative risks between the 2 groups as odds ratios (ORs) and 95% confidence intervals (CIs). Our first step was to estimate the heterogeneities between studies. Heterogeneity between studies was estimated using the *I^2^* statistic, with *I* ^2^ above 50% reflecting significant heterogeneity. If the results of the heterogeneity analysis were significant, we used random effect models to combine the results by including random effects and we used fixed effect models to conduct meta-analysis oppositely when *I^2^* <50 *(* 18).

To assess the impact of individual studies on this meta-analysis, sensitivity analyses were performed by excluding 1 dataset at a time. In addition, publication bias was estimated by constructing funnel plots based on visual judgements of the symmetry of the funnel plots. We conducted the above analysis using R v4.1.2 and the ‘meta’ R package.

## Results

### Study search

**Figure 1:**
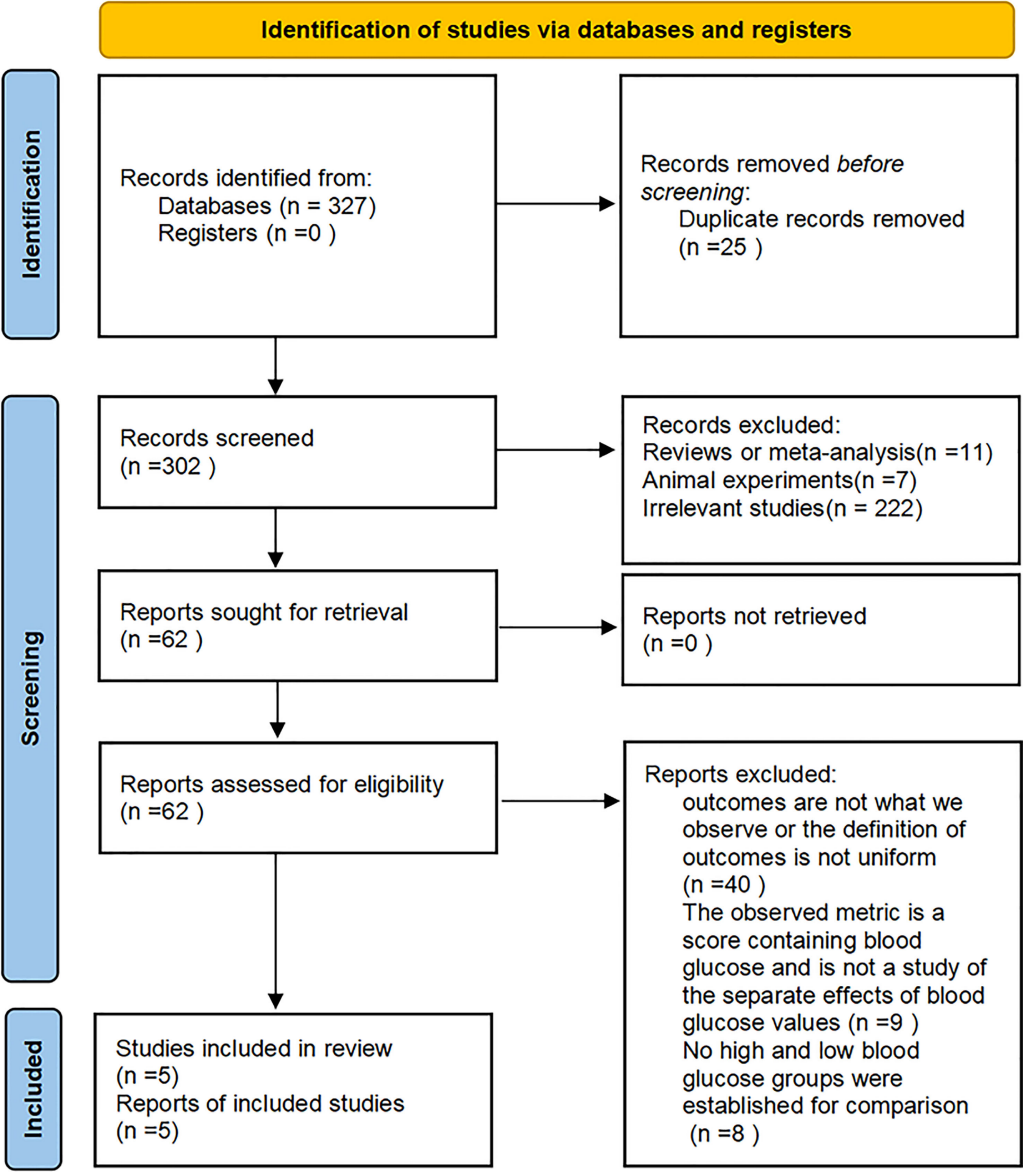
Flowchart of study search.

A flowchart of the literature search and study inclusion procedure is presented in **Figure 1**. We found 327 articles in our initial database search. After eliminating 25 duplicates, we screened 302 studies left based on their titles and abstracts. Eleven studies were excluded mainly because they were reviews or meta-analysis, 7 articles were excluded because they were animal experiments, and 222 researches were excluded because they were not relevant to the objective of our meta-analysis. In the end, 62 studies were reviewed in full-text, and 57 were excluded for the reasons listed in **Figure 1**. Finally, 5 studies were included in this meta-analysis.

### Study characteristics

Five studies were included in the meta-analysis and characteristics of these studies were presented in **Table 1** (6, 8, 19–21). In all studies, stroke patients received intravenous tissue plasminogen activator, with mean ages ranging from 67 to 73 years old. Random blood glucose values at admission were measured, and the definitions of hyperglycemia were consistent across studies or varied so little that they could be put together for statistical analysis. The duration of observation and definition of the outcome indicators and scores for poor clinical outcome were consistent across the studies. What’s more, the NOS for all included studies was 8 to 9 stars, indicating good study quality (**Table 2**).

**Table 1:** Characteristics of the included cohort studies.

Study	Contries	Design	Diagnosis	Definition of hyperglycemia	Mean age(years)	Male (%)	Outcome events
Alvarez-Sabín, 2003	Spain	prospective	IV-tPA–treated stroke patients	Hyperglycemia was defined as a blood glucose level >140 mg/dL(7.7mmol/l)	70.4	49.3	sICH;The modified Rankin Scale>2 at 90 days.
Poppe,2009	Canada	prospective	IV-tPA–treated stroke patients	Hyperglycemia was defined as a glucose level >144mg/dl (8.0mmol/l)	73	55	sICH, functional outcome at 90 days, and death
Putaala, 2011	Finland	retrospective	acute ischemic stroke treated with intravenous thrombolysis	Hyperglycemia was defined as a blood glucose level of >144mg/dl (8.0 mmol/l).	70	55.2	unfavorable 3-month outcome (mRS>2), death, and sICH according to NINDS criteria.
Yaghi, 2012	America	prospective	IV-tPA–treated stroke patients	Hyperglycemia was defined as a blood glucose level of > 144mg/dl (8.0mmol/l)	67.2	55	sICH, and outcome at 3 months defined by mRS.
Saqqur, 2015	Canada	retrospective	IV-tPA–treated stroke patients	Hyperglycemia was defined as a glucose level ≥140 mg/dl (7·7 mmol/l).	68.4	54.6	poor clinical outcome (3month mRS > 2), sICH

IV, intravenous injection; tPA, tissue plasminogen activator;

ICH, intracranial hemorrhage; sICH, symptomatic intracerebral hemorrhage;

NINDS, National Institute of Neurological Disorders and Stroke

**Table 2:** Quality evaluation of the included cohort studies *via* the NOS.

Study	Representativeness of the exposed cohort	Selection of the non-exposed cohort	Ascertainment of exposure	Outcome not present at baseline	Control for age and sex	Control for other confounding factors	Assessment of outcome	Enough long follow-up duration	Adequacy of follow-up of cohort	Total
Alvarez-Sabín, 2003	1	1	1	1	1	1	1	1	0	8
Poppe, 2009	1	1	1	1	1	1	1	1	0	8
Putaala, 2011	1	1	1	1	1	1	1	1	1	9
Yaghi, 2012	1	1	1	1	1	1	1	1	1	9
Saqqur, 2015	1	1	1	1	1	1	1	1	1	9

NOS: Newcastle-Ottawa Scale.

### Higher blood glucose levels increase the risk of symptomatic intracranial hemorrhage

**Figure 2:**
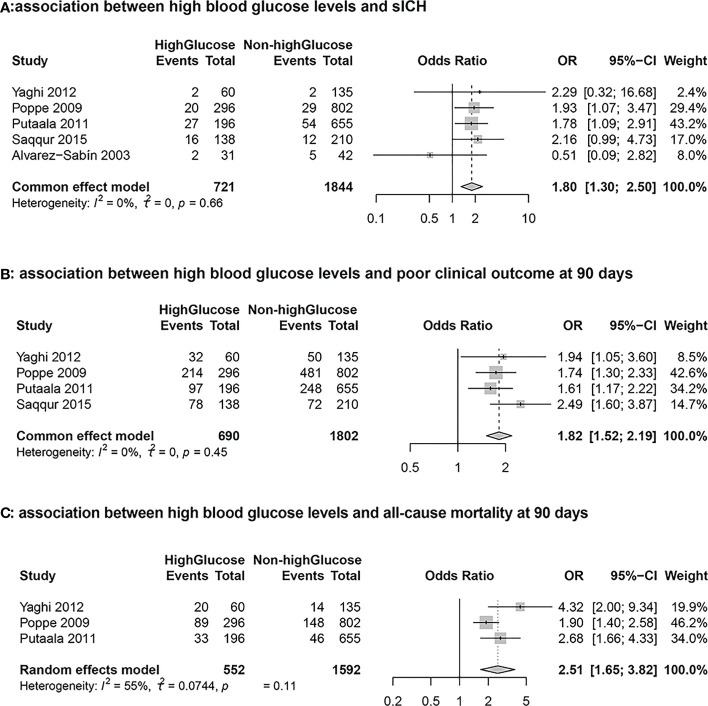
Forest plots for the meta-analyses of the outcomes between high glucose and non-high glucose groups. **(A)**: Association between hyperglycemia and symptomatic intracranial hemorrhage (sICH) **(B)**: Association between hyperglycemia and poor clinical outcome at 90 days **(C)**: Association between hyperglycemia and all-cause mortality at 90 days.

**Figure 3:**
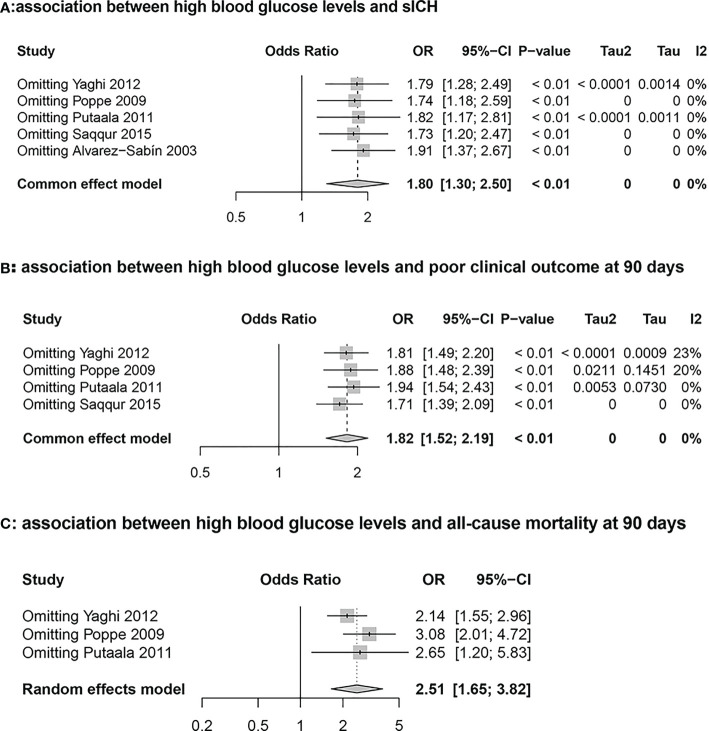
Sensitivity analysis graph for the high glucose and non-high glucose groups. **(A)**: Association between hyperglycemia and symptomatic intracranial hemorrhage (sICH) **(B)**: Association between hyperglycemia and poor clinical outcome at 90 days **(C)**: Association between hyperglycemia and all-cause mortality at 90 days.

Five cohort studies including 2565 stroke patients who received intravenous tissue plasminogen activator evaluated the association between hyperglycemia and sICH. During the follow-up period, a total of 169 patients developed sICH. The results from the meta-analysis indicated that blood glucose level at admission was independently associated with a higher risk of sICH (High Glucose *vs* Non-high Glucose, OR: 1.80, 95% CI: 1.30-2.50, I2 = 0%; **Figure 2A**). The results from sensitivity analyseswere consistent (overall OR: 1.80; 95%CI: 1.30-2.50; p <0.01; **Figure 3A**).

### Higher blood glucose levels increase the risk of poor clinical outcome at 90 days

Four studies including 2492 stroke patients who received intravenous tissue plasminogen activator evaluated the association between blood glucose level and poor clinical outcome. A total of 1272 patients had 90-day mRS scores > 2 during the follow-up period. The results from the meta-analysis indicated that blood glucose level at admission was independently associated with a higher risk of 90-day mRS scores > 2 (High Glucose *vs* Non-high Glucose, OR: 1.82, 95% CI: 1.52-2.19, I2 = 0%; **Figure 2B**). The results from sensitivity analyses, which excluded one data set at a time, were consistent (OR: 1.82 95%CI:1.52-2.19, p all <0.0; **Figure 3B**).

### Higher blood glucose levels increase the morality at 90 days

Three cohort studies including 2144 stroke patients who received intravenous tissue plasminogen activator evaluated the association between hyperglycemia and all-cause morality. A total of 350 patients died during the follow-up period. The results from the meta-analysis indicated that blood glucose level at admission was independently associated with a higher risk of death (High Glucose *vs* Non-high Glucose, OR: 2.51, 95% CI:1.65-3.82, I2 = 55%; **Figure 2C**). The results from sensitivity analyses, which excluded one data set at a time, were consistent. (OR: 2.51 95%CI:1.65-3.82; **Figure 3C**).

### Publication bias

**Figure 4:**
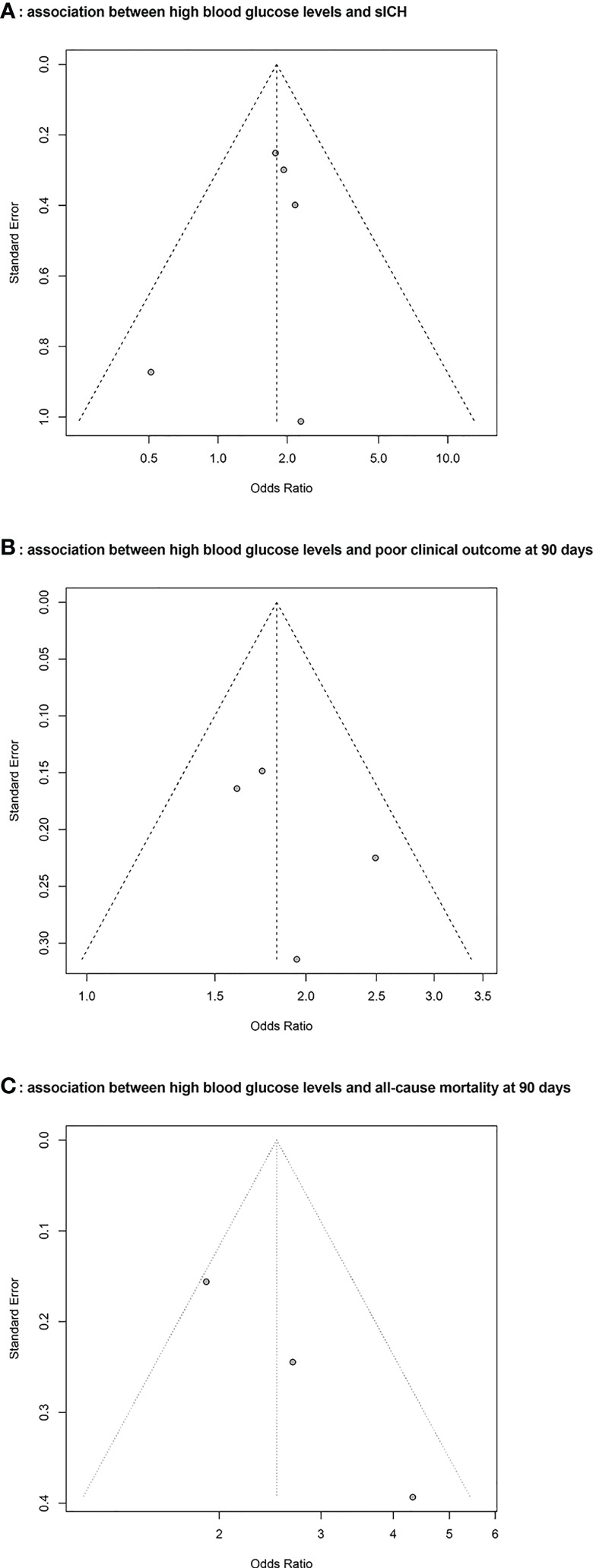
Funnel plots for the publication bias underlying the meta-analyses. **(A)**: Association between hyperglycemia and symptomatic intracranial hemorrhage (sICH) **(B)**: Association between hyperglycemia and poor clinical outcome at 90 days **(C)**: Association between hyperglycemia and all-cause mortality at 90 days.

These funnel plots show the association between blood glucose level and poor clinical outcomes in AIS patients receiving intravenous tissue plasminogen activator **Figure 4**. According to visual inspection, the plots were symmetrical, indicating a low risk of publication bias.

## Discussion

Currently, IVT is an essential and widely used method of treatment for AIS. In order to illustrate the relationship between blood glucose level and prognosis of AIS patients who received intravenous thrombolysis, we specifically searched for 5 studies and conducted a meta-analysis on this topic.

The increased risk of sICH after intravenous thrombolysis in high glucose level group compared with non-high glucose level points to an effect of blood glucose levels on the risk of sICH. Previous studies have also shown that admission blood glucose is one of the predictors of sICH (22–24), even after controlling for HbA1c (25). Possible mechanisms include higher blood glucose impairing cellular metabolism, reducing vascular reactivity, increasing blood-brain barrier permeability and exacerbating acidosis in reperfused brain tissue. As well, the prevalence of atherosclerosis is also higher in the diabetic population compared to the non-diabetic population. This can indirectly increase the risk of sICH after thrombolysis. Thirdly, from a pathophysiological point of view, studies have established a rat model of stroke, which proved that alteplase treatment increased cerebral hemorrhage after stroke, and blood-brain barrier (BBB) leakage increased (26, 27). IVT may further aggravate BBB leakage by destroying (28).

Among patients with poor clinical outcomes, the high glucose group also showed a more significant risk than the non-high glucose group. The mRS is a measure of a patient’s functional recovery after a stroke and is graded from 0 to 6. Therefore, in this study we defined poor clinical outcome as mRS > 2 (29). There are studies suggesting that high blood glucose level affects the thrombolytic effect of alteplase and its effect on the evolution of cerebral infarction (7). On this issue, studies have also confirmed that the deep hemispheric white matter is part of the clinically relevant penumbra and shown that hyperglycemia exacerbates the appearance of irreversible ischemic damage in this region within 24 hours (30). This may be one of the mechanisms contributing to poor clinical outcomes. In addition, it has also been shown in recent years that higher blood glucose levels stimulate the thrombophilia cascade response, which amplifies Downstream microvascular thrombo-inflammation (DMT) caused by middle cerebral artery occlusion. Then DMT exacerbates the damage to reperfusion and precipitates a range of functional and structural neurological impairments (31, 32). Therefore, irreversible damage to neurological function and structure caused by hyperglycemia can have adverse clinical outcomes. On the clinical side, there are also studies that have statistically shown a high rate of combined hyperglycemia in stroke patients with the potential to affect long-term outcomes (33). Therefore, these may be clinically relevant mechanisms that are hypothesized to explain why hyperglycemia exacerbates adverse clinical outcomes.

As for mortality, it is clear from our analysis that patients with AIS with higher blood glucose levels are also at greater risk of death after receiving intravenous tissue plasminogen activator. In addition to the neurological impairment mentioned above that may lead to death, patients with higher blood glucose levels are also prone to infection, which is a possible cause of death for patients with AIS after intravenous thrombolysis who need to stay in bed for a long time. Therefore, blood glucose levels should be assessed in AIS patients receiving intravenous tissue plasminogen activator for risk stratification and clinical decision-making to reduce mortality.

Furthermore, the higher prevalence of co-morbidities in the high glucose group may be due to older age and a higher proportion of diabetes. When patients were classified according to whether they had admission hyperglycemia, patients with high glucose were more likely to have hypertension, hyperlipidemia, coronary artery disease, and known diabetes. Concerning the covariate factors affecting the prognosis of patients after thrombolysis, our initial study showed that increasing age, history of diabetes, admission glucose ≥140 mg/dL, and early embolism were associated factors of poor clinical outcomes in reperfused patients. However, logistic regression models showed that only admission glucose ≥140 mg/dL emerged as an independent predictor of poor clinical outcome (6). According to previous studies, the odds of having a sICH increase with increasing blood glucose on admission (34). Therefore, it is reasonable to speculate that extremely high blood glucose would produce a different clinical outcome than moderate high levels of blood glucose.

Although we rigorously conducted data retrieval, screening, and analysis, the following limitations of our study remain. Firstly, three of the studies included in the meta-analysis were retrospective. Data collection in retrospective studies is not subject to investigator control and assessment and may result in some bias. Secondly, regarding cut-off values for the high and non-high glucose groups, we had 2 primary studies defined 140 mg/dl and the other three primary studies defined 144 mg/dl. This may contribute to heterogeneity. Each original study referred to the American Diabetes Association criteria of hyperglycemia at admission or previous relevant studies to develop cut-off values for the high blood glucose and non-high blood glucose groups (10, 35, 36). The definitions of hyperglycemia are very close between the various studies, and there are further studies that suggest that a glucose level of approximately 140 mol/dl may indicate a watershed level and that patients with glucose above this cut-off have worse clinical outcomes (37, 38). Since there was no significant difference in the cut-off values of blood glucose among the studies, for the purpose of data analysis, we used 140 mg/dl as the cut-off value for the blood glucose group in this meta-analysis. Thirdly, In the analysis regarding mortality, we found heterogeneity between the studies through the test of heterogeneity. Regarding the heterogeneity, in addition to the possible reasons mentioned above, another possible reason is that the sample size of one study is a little small compared to the other 2 studies. Therefore, we used a random effects model to estimate the combined effect size for the data of this outcome to partially correct for meta-analysis heterogeneity in order to improve the precision of the estimated confidence intervals and to increase the test efficacy at the same time. Moreover, our study did not include grey literature such as conference abstracts, which may have led to an incomplete analysis. In addition, we did not observe whether the fluctuation of blood glucose level during hospitalization would have an impact on AIS after intravenous thrombolysis, which may lead to a lack of rigorous analysis. In addition, considering that the original study we included was based only on a single admission randomized glucose value and did not measure glycated hemoglobin in patients, this may have led to less comprehensive monitoring of our blood glucose. This is an issue that needs to be improved in further studies.

Based on the results of 5 studies, we found that higher blood glucose level could have a poor prognosis of AIS patients treated with intravenous tissue plasminogen activator. The results of the sensitivity analysis were consistent one data set at a time. In conclusion, these results suggest that hyperglycemia may be a useful predictor of sICH, poor clinical outcome and all-cause mortality in stroke patients who received intravenous tissue plasminogen activator.

## Conclusion

High blood glucose level is an important clinical consideration for prognosis in patients with AIS who receive intravenous thrombolytic therapy. Our meta-analysis showed that high blood glucose levels were closely associated with sICH, poor clinical outcomes, and mortality following intravenous thrombolysis.

## Data availability statement

The original contributions presented in the study are included in the article/supplementary materials. Further inquiries can be directed to the corresponding authors.

## Author contributions

JHZ, YW and JZ contributed to conception and design of the study. YW organized the database. GJ performed the statistical analysis. YW wrote the first draft of the manuscript. YW, GJ and JZ wrote sections of the manuscript. All authors contributed to manuscript revision, read, and approved the submitted version.
